# The aryl hydrocarbon receptor in liver inflammation

**DOI:** 10.1007/s00281-021-00867-8

**Published:** 2021-06-01

**Authors:** Antonella Carambia, Fenja Amrei Schuran

**Affiliations:** Department of Medicine I, University Medical Center Hamburg-Eppendorf, Martinistr. 52, D-20246 Hamburg, Germany

**Keywords:** Aryl hydrocarbon receptor, AHR ligands, Hepatic immune response, Hepatic tolerance, Liver inflammation, Therapy

## Abstract

The aryl hydrocarbon receptor (AHR) is a ubiquitously expressed ligand-activated transcription factor with multifaceted physiological functions. In the immune system, AHR has been unequivocally identified as a key regulatory factor that can integrate environmental, dietary, or microbial signals into innate and adaptive immune responses. Correspondingly, AHR activity seems to be most important at barrier organs, such as the gut, skin, and lung. The liver is likewise prominently exposed to gut-derived dietary or microbial AHR ligands and, moreover, generates plenty of AHR ligands itself. Yet, surprisingly little is known about the role of AHR in the regulation of hepatic immune responses, which are normally biased towards tolerance, preventing harmful inflammation in response to innocuous stimuli. In this review, we summarize the current knowledge about the role of AHR in hepatic immune responses in the healthy liver as well as in inflammatory liver disease. Moreover, we discuss AHR as a potential therapeutic target in hepatic disorders, including autoimmune liver disease, liver fibrosis, and liver cancer.

## Hepatic immune responses

In the healthy liver, immune responses normally result in tolerance rather than inflammation. This bias towards hepatic tolerance is of paramount importance since the liver is steadily exposed to harmless but potentially immunogenic compounds, such as nutritional or microbial antigens reaching the liver via the portal blood [1, 2]. To maintain immune homeostasis, liver non-parenchymal cells (NPCs), including dendritic cells (DCs), Kupffer cells (KCs), or liver sinusoidal endothelial cells (LSECs), function as tolerogenic scavenger and antigen-presenting cells (APCs) that regulate innate as well as adaptive immune responses. Indeed, liver NPCs have an important function in protecting the organism by clearing portal blood from gut-derived endotoxin which requires them to tolerate high endotoxin doses without releasing inflammatory cytokines. Furthermore, liver APCs promote tolerogenic CD4 as well as CD8 T cell responses to presented antigens [1, 2]. For example, LSECs can suppress inflammatory Th1 and Th17 responses [3], induce IL-10-producing CD4+ T cells [4] and FOXP3+ regulatory T cells [5], or tolerize CD8+ T cells dependent on PD-L1 [6]. Likewise, KCs have been shown to suppress CD4+ T cell responses dependent on prostaglandins [7]. Yet, hepatic tolerance and suppression of inflammation may come at the cost of increased susceptibility for liver infection or cancer [1, 2]. Moreover, immune homeostasis can be perturbed by various extrinsic factors, including environmental toxins, drugs, diet, infections, or alterations in the gut microbiome [1, 8, 9], resulting in liver disease.

The development of new therapeutic interventions that either preserve hepatic immune homeostasis or boost anti-infectious or anti-tumor immunity presupposes a more detailed understanding of the regulatory mechanisms underlying hepatic immune responses. In this article, we provide an overview of the current knowledge about the role of AHR in the regulation of hepatic immune responses in the healthy and diseased liver. Moreover, we discuss the potential of AHR as a new therapeutic target in liver disease.

## AHR activation and signaling

**Fig. 1:**
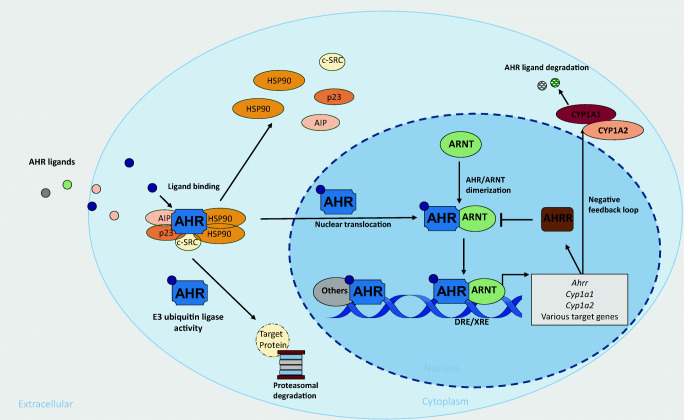
AHR signaling pathway. The inactive AHR is complexed with the chaperon HSP90, co-chaperon p23, AIP, and c-SRC in the cytoplasm. Ligand binding results in conformational changes of AHR, dissociation of the protein complex, and AHR translocation into the nucleus. In the nucleus, AHR forms a heterodimer with ARNT. AHR/ARNT binds to dioxin/xenobiotic responsive elements (DRE/XRE), inducing transcription of various target genes. Additionally, in complex with other transcription factors, AHR can interact with alternative binding sites. AHR activation is limited in a negative feedback loop by the AHR repressor AHRR inhibiting AHR/ARNT dimer formation and by the AHR-induced enzymes CYP1A1 and CYP1A2 which degrade AHR ligands. Besides its transcriptional activity, AHR also functions as part of the E3 ubiquitin ligase complex driving the proteasomal degradation of target proteins, most notably of hormone receptors

AHR is a ligand-activated transcription factor of the evolutionarily conserved Per-Arnt-Sim (PAS) superfamily, acting as sensors of various environmental signals. In its inactive state, AHR resides in the cytoplasm as part of a protein complex (including HSP90, p23, c-SRC, and AHR interacting protein), protected from degradation and translocation into the nucleus. Ligand binding induces conformational changes of AHR leading to dissociation of the protein complex and nuclear translocation. In the nucleus, together with its binding partner AHR nuclear translocator (ARNT), AHR can induce transcription of various target genes featuring dioxin-responsive elements (DREs) as AHR-ARNT binding sites [10, 11]. In a negative feedback loop limiting AHR activation, degradation of AHR ligands is facilitated by cytochrome P450-dependent monooxygenases CYP1A1 and CYP1A2, which are directly activated by AHR. Moreover, the AHR repressor (AHRR), a direct downstream target of AHR, also confines AHR signaling [10, 11]. Of note, besides binding to DREs, in complex with other transcription factors, AHR can also be recruited to other target sequences. For instance, together with RELA and RELB, AHR can bind to NF-κB responsive elements [12]. Moreover, AHR can also influence biological processes in a non-genomic manner. Dissociation of the cytoplasmic AHR complex upon ligand binding releases several biologically active molecules such as the c-SRC protein kinase, leading to phosphorylation of multiple target genes [12]. Interestingly, AHR has also been identified as an adaptor protein of the ubiquitin ligase complex, determining substrate specificity for proteasomal degradation of steroid receptors, such as the estrogen receptor [12, 13], or the Peroxisome proliferator-activated receptor γ (PPARγ), a central regulator of adipogenesis [14]. Figure 1 provides a schematic overview of the AHR signaling pathway.

## AHR ligands

Originally, AHR had been recognized as receptor for dioxin and other pollutants and xenobiotics [11, 15]. Yet, during the last decade, a huge and still growing number of additional non-toxic nutrient-derived and endogenously generated AHR ligands have been identified. Importantly, recent studies have linked the bioavailability of particular AHR ligands, including indole derivatives of tryptophan, to the gut microbiota composition [11, 15]. Moreover, it has become evident that AHR ligands play a major role in regulation of innate as well as adaptive immune responses. Therefore, as a sensor of environmental cues, AHR can integrate signals from toxins, dietary metabolites, or microbial compounds into the immune response [11, 12, 15–17]. Yet, the actual outcome of AHR activation is determined by the respective AHR ligands that not only differ in origin, but also AHR affinity and half-life. Furthermore, the regulation of immunologically relevant genes by AHR critically depends on the contextual signals in the target cell, as AHR can form cell type-dependent transcriptional complexes with various other molecules [12]. A thorough overview of known AHR ligands and their respective impact on the immune system is depicted in references 10–12. Herein, we focus specifically on AHR ligands described in the context of hepatic immune responses (Table 1).

**Table 1:** Selected AHR ligands and their roles in liver disease

AHR ligand	Origin	Ahr affinity	Impact on liver homeostasis
TCDD (=dioxin) (2,3,7,8-tetrachlorodibenzo-*p*-dioxin)	Exogenous Environmental pollutant	High No bio-degradation	Dampens ConA-induced hepatitis via myeloid-derived suppressor cells [38] TCDD-exposed DCs from PBC patients promote inflammatory Th1 and Th17 differentiation [44] Interferes with host resistance to *T. cruzi* infection due to impaired effector T cell subsets [46] Repression of cytokine-induced acute phase genes in primary hepatocytes [47] Induces liver fibrosis, hepatotoxicity, and inflammation [31] Increases necroinflammation and hepatic stellate cell activation but not hepatic fibrosis[56]
ITE 2-(1'H-indole-3'-carbonyl)-thiazole-4-carboxylic acid methyl ester	Host metabolism Tryptophan derivative	High	ITE + 3-HK: Induction of Treg cells, abolishes protective immunity against *T. cruzi* infection [46] Exacerbation of acetaminophen-induced liver injury via Cyp1a2 induction in hepatocytes [52] Inhibition of HSC activation and prevention of CCl4 induced liver fibrosis [54]
FICZ (6-Formylindolo[3,2-b]carbazole)	Host metabolism UV photo-oxidation of tryptophan	High	Exacerbation of acetaminophen-induced liver injury [52] Inhibits IFN-γ production by hepatic NKT cells in ConA mediated liver injury [34] Reduces alcohol induced liver pathology and increases anti- microbial peptide levels in the gut [49]
Kynurenine 3-HK (3-hydroxy-kynurenine)	Host metabolism Tryptophan derivatives	Low	Kynurenines produced by IDO1 in HSCs in response to LPS challenge enhance suppressive capacity of nTregs [33] Exacerbation of CCl4-induced acute liver injury upon blockade of Kynurenine-producing IDO2 [57] ITE + 3-HK: Induction of Treg cells, abolishes protective immunity against *T. cruzi* infection [46]
I3C (Indole-3-carbinol)	Dietary Glucobrassicin-derived Ahr ligand precursor	Low	Preventive in alcohol-induced liver injury [50]
DIM (3′3-diindolylmethane)	Dietary I3C metabolite	High	Ameliorates experimental hepatic fibrosis by downregulation of miR-21 expression [55] Reduction of hepatic steatosis and progression of NASH by reversing Th17/Treg imbalance to Treg predominance [63]
ß-NF (β-naphthoflavone)	Dietary	Moderate	Attenuation of cytokine-mediated acute-phase response *in vivo* [47]
I3A (indole-3-acetate)	Microbiome Tryptophan derivative	Low	Inhibition of inflammatory cytokine expression in macrophages in response to LPS and fatty acids [48] Inhibition of inflammatory hepatocyte activation in response to TNF-a and fatty acids [48]
Tryptamine	Microbiome Tryptophan derivative	Low	Inhibition of inflammatory cytokine expression in macrophages in response to LPS and fatty acids [48]

**Table 2:** Cell type specific regulatory effects of AHR in liver disease

Cell type	AHR ligand	AHR target genes	Disease-promoting (+) or disease-attenuating (-) AHR effects	Use of cell-spec. *Ahr* KO	Ref.
Hepatocytes	ß-NF	NFκB	Acute phase response (-)	Yes	[47]
	I3A	Fasn, SREBP-1c	Lipogenesis (-)	No	[48]
	ITE, FICZ	Cyp1a2	APAP hepatotoxicity (+)	Yes	[52]
	FICZ [49], I3C [50]	Cyp1a1 [49], Scd1 [49]	Alcohol-induced liver injury (-)	Yes	[49,50]
	Dioxin	Cyp1a1, Cyp1a2, Cyp1b1	Hepatotoxicity (+)	Yes	[27]
	TCDD	Cyp1a2, CD36	Steatosis (+)	No	[58]
	ANF	Cyp1a1, TNF-α	Oxidative stress (+), insulin resistance (+), NAFLD (+)	No	[59]
	BaP	Cyp1a1	Estrogen degradation (+), steatosis (+)	No	[60]
	TCDD	Fgf21	Steatosis (+), systemic insulin hypersensitivity (+)	No	[61]
	3MC	Socs3	Lipogenesis (-), steatosis (-), liver inflammation (-) under HFD	Yes	[62]
	Kyn	Cyp1a1	IDO2 induction, CCl4-induced acute liver injury (+)	No	[57]
Endothelial cells	Unknown	Unknown	Failure of ductus venosus closure, immature sinusoidal architecture, portal hypertension	Yes	[27]
HSCs	Kyn	Cyp1b1	Kynurenine-derived from HSC fosters Treg expansion and function	No	[33]
	ITE	β-Catenin, Smad3	HSC activation (-), fibrogenesis (-)	Yes	[54]
Macrophages	I3A, tryptamine	TNF- α, IL-1β, MCP-1	Inflammatory mediators (+)	Yes	[48]
DCs (PBC patients)	TCDD	Cyp1a1	Induction of Th1 and Th17 response (+)	Human	[44]
Invariant NKTs	FICZ	IFN-γ	Tissue residency (+), IFN-γ production (-), ConA hepatitis (-)	No	[34]
MDSCs	TCDD, 3MC	CXCR2, miR-150-5p, miR-543-3p	MDSC induction, ConA hepatitis (-)	No	[38]
ILCs	FICZ	IL-22	IL-22 induction, ConA hepatitis (-)	No	[34]
CD4+ T cells	N2ICD [35], miR15a/16-1 [37]	IL-22 [35,37], Cyp1a1 [35]	IL-22 induction, ConA hepatitis (-)	No	[35, 37]
Tregs, Th17	UCB, quercetin, Kyn	Cyp1a1, CD39, ER-α, HIF1-α	Impaired AHR and CD39 expression correlates with AIH severity	Human	[41]
	DIM	Cyp1a1, Cyp1b1	Treg (+), Th17 (-), inflammation (-), steatosis (-) under MCD-diet	No	[63]
	Kyn	ISX	TDO (+), IDO1 (+), PD-L1 (+), CD8 T cell response (-), tumor cell proliferation (+) in HCC	Human	[66]

## AHR immune function

As there are several recent reviews excellently summarizing the role of AHR as a key regulatory molecule in the immune system [10–12, 16–18], the impact of AHR on immune cells is only briefly discussed here.

AHR activity has been shown to impact on a variety of innate and adaptive immune cells, including innate lymphoid cells (ILCs), T cells, B cells, DCs, and macrophages. Notably, depending on the particular cell type and contextual signals, AHR may have both pro-inflammatory and tolerogenic functions. Regarding ILCs, AHR is particularly important for the differentiation and maintenance of ILC3s, the innate counterpart of Th17/Th22 cells. Indeed, *Ahr* knockout mice have very few IL-22 producing ILC3s, resulting in increased susceptibility to bacterial infection and colitis. Thus, by fostering IL-22 production, AHR is an important regulator of immune homeostasis in the gut [19, 20]. Likewise, AHR critically influences NK cell function; upon ligand-dependent activation, AHR promotes anti-tumor cytotoxicity as well as INFγ production [21]. Moreover, tissue residency of hepatic NK cells has been linked to AHR signaling [22].

In T cells, AHR can have pro- or anti-inflammatory functions. While promoting the differentiation of inflammatory IL-22-producing Th17 cells, AHR is also critically involved in the induction of regulatory T cell populations such as CD4+CD25+Foxp3+ Treg or Tr1 cells [12]. In APCs, AHR has been associated with tolerance-promoting activities; indeed, in DCs, AHR can induce down-regulation of MHC II and co-stimulatory molecules and suppression of Th1- and Th17-polarizing cytokines [12]. Moreover, in DCs, AHR can induce the expression of indolamine-2,3-dioxygenase (IDO) and secretion of retinoic acid (RA), important mediators of Treg differentiation [12]. For macrophages, it has been shown that together with the transcription factor c-Maf, AHR promotes the expression of the anti-inflammatory cytokine IL-10 [12]. Likewise, as a key mediator of endotoxin tolerance [23], AHR constrains the inflammatory IL-6 and TNF response to LPS in macrophages [24].

## AHR in liver immune homeostasis

According to its function as sensor of environmental cues, *Ahr* is highly expressed in barrier organs such as the gut, skin, and lung, as well as in the liver [17]. However, whereas AHR has been unequivocally identified as a key regulatory factor in mucosal immune regulation, the immunological relevance of AHR in the liver is only beginning to be elucidated. Nonetheless, there are several indications that AHR might regulate immune responses within the liver. Note that AHR activity is crucial for normal liver development, as reflected by decreased liver and hepatocyte size, impaired polyploidization, an immature sinusoidal architecture, the failure of neonatal ductus venosus closure, and considerable portosystemic shunting in *Ahr* knockout mice [25–27]. *Ahr* knockout mice carrying a deletion of exon 1 develop a liver pathology characterized by portal fibrosis and biliary inflammation already at the age of 3 weeks; another *Ahr* knockout strain with deletion of exon 2 seems to develop mild cholangitis and portal fibrosis only at later time-points [28–30]. The reasons for the observed phenotypical differences between *Ahr* knockout strains are not clear but might rely on several variables, including the respective gene targeting strategy that may result in altered gene products with unsuspected function or that may affect neighboring genes, as well as the type of embryonic stem cells used, or the genetic background of recipients [28]. Moreover, it has been shown that the treatment of wild-type mice with the high-affinity ligand TCDD (dioxin) leads to liver inflammation and fibrosis [31]. Regarding AHR-dependent immune regulation in the liver, it is of particular interest that tryptophan-derived tolerogenic AHR ligands, the kynurenines, are constitutively produced within the liver by the hepatocyte-specific enzyme tryptophan-2,3-dioxygenase (TDO2) [32]. Kynurenine-mediated AHR activation has been reported to induce immunosuppression in both T cells and APCs [12]. Likewise, in response to LPS challenge, induction of kynurenine-producing IDO1 in hepatic stellate cells (HSCs) leads to enhanced AHR signaling in thymus-derived natural Tregs, which in turn induces up-regulation of Foxp3, epigenetic stabilization, expansion, and enhanced suppressive capacity [33]. Moreover, an interesting function in the maintenance of a newly discovered population of liver-resident NK cells has been ascribed to AHR. Indeed, *Ahr* knockout mice display reduced numbers of these CD49a+TRAIL+CXCR6+DX5-liver-resident NK cells, most likely depending on increased susceptibility to cytokine-induced cell death [22].

## AHR in immune-mediated liver disease

The role of AHR in acute immune-mediated liver injury has been investigated in the Concanavalin (Con)A-model in several studies, establishing a protective function of AHR in acute immune-mediated hepatitis. Indeed, in bone marrow chimera experiments in which hematopoietic cells from *Ahr* knockout mice were adoptively transferred to irradiated wild-type recipients, Abe et al. demonstrated that increased susceptibility to ConA-mediated liver injury was due to an uncontrolled IFN-γ response from invariant NKT cells along with decreased production of tissue-protective IL-22 by ILCs [34]. The crucial role of AHR in the induction of IL-22 and protection from immune-mediated liver injury was further linked to Notch signaling. Notably, Notch induced the expression of endogenous AHR ligands and IL-22 in CD4+ T cells, which in turn fostered AHR-dependent induction of protective IL-22 [35]. Along these lines, a second study from the same group investigating the impact of IL-23 in acute liver injury confirmed a regulatory role of AHR by fine-tuning pro-inflammatory IL-17 as well as protective IL-22 responses [36]. Furthermore, the repression of AHR-dependent IL-22 expression in CD4+ T cells by microRNA 15a/16-1 led to exacerbated liver inflammation following ConA challenge, confirming the key role of AHR in controlling inflammatory responses in acute liver injury induced by ConA [37]. Interestingly, in a recent study, AHR has also been implicated in induction and mobilization of highly immunosuppressive myeloid-derived suppressor cells (MDSCs) from the peritoneal cavity. Accordingly, in adoptive transfer experiments, MDSCs induced by the AHR ligand TCDD dampened ConA-induced hepatitis [38].

There is increasing evidence that impairment of AHR might also be involved in the pathogenesis of immune-mediated liver diseases in humans. It is likely that the dual function of AHR in supporting either tolerogenic or inflammatory T cell responses, i.e., for example through induction of Treg or Th17 differentiation, might be important for hepatic immune regulation. Indeed, higher frequencies of Th17 cells and Treg impairment are implicated in the pathogenesis of autoimmune liver diseases [39, 40]. Recently, Vuerich et al. investigated the impact of AHR signaling on the surface expression of CD39 on Th17 and Treg cells in autoimmune hepatitis (AIH) [41]. In their former studies, the same group suggested that impaired expression of CD39, an ATP-degrading ectoenzyme that fosters the production of immunosuppressive adenosine, both, on Treg and Th17 cells is a major cause of autoimmune pathology in AIH [42]. Here, the authors suggest a link between defective AHR signaling and loss of CD39 expression and function in Tregs and Th17 cells from AIH patients. Indeed, upon AHR stimulation in vitro, Tregs and Th17 cells derived from AIH patients showed less CD39 expression and activity as well as decreased Treg suppressor function in comparison to Tregs and Th17 cells from healthy donors. Loss of immune homeostasis in AIH patients with impaired AHR activity was attributed to increased expression of the AHR repressor AHRR and high levels of HIF-1α, a competitive binding partner for ARNT, thereby inhibiting AHR signal transduction and consequently CD39 in Tregs and Th17 cells in human AIH. Furthermore, the authors observed preferential binding of AHR to the estrogen receptor ERα rather than to ARNT, fostering non-canonical AHR signaling instead of CD39 expression relying on signal transduction via AHR/ARNT [41]. In autoimmune cholangitis, analysis of peribiliary infiltrates in human liver tissue demonstrated the presence of CCR6+ CD4+ and AHR+ CD4+ T cells that could potentially develop into Th17 cells under the influence of biliary epithelial cell-derived IL-1 and IL-6 and the CCR6 ligand CCL20 [43]. Regarding primary biliary cholangitis (PBC), there is emerging evidence that AHR might be involved in dysregulation of T cell responses. Consistent with the notion that AHR-activating dioxins might be risk factors for the development of autoimmune disease [44], She et al. reported that DCs derived from PBC patients but not from healthy donors enhanced inflammatory Th1 and Th17 differentiation upon TCDD exposure [44]. In dnTGFβRII-deficient mice, a spontaneous model for PBC, microarray analysis linked dysfunctional Treg showing reduced suppressive capacity and acquisition of inflammatory features to the down-regulation of AHR and other critical transcription factors in Tregs. Therefore, the authors suggest further analysis of potentially aberrant signaling pathways, including the AHR pathway in Tregs from human PBC patients [45].

Interestingly, AHR has also been implicated as immunoregulatory factor in liver infection. Indeed, *Trypanosoma cruzi*-infected *Ahr*d mice, which display a mutant AHR with low ligand affinity, displayed low numbers of Tregs, an effective Th1 response, development of CD8+ T cell memory subsets, and consequently low parasite burden. Of note, the concomitant induction of systemic IL-10 constricted the anti-infectious immune response and prevented liver immunopathology [46].

Notably, also the acute phase response in the liver seems to depend on AHR. Patel et al. showed that via repression of the NF-κB pathway, AHR regulates the expression of cytokine-induced acute phase genes such as SAA1/2. Likewise, dietary supplementation with AHR ligands resulted in down-regulation of the acute phase response in vivo. Therefore, the authors suggest a key role of AHR in regulation of liver inflammation that might be targeted therapeutically [47].

In conclusion, the currently available literature suggests that depending on the respective disease, the particular cell type, and the activating ligands, AHR seems to have predominantly anti-inflammatory and tissue-protective function in immune-mediated liver disease.

## AHR and the gut-liver axis

As described above, the gut microbiome is a major source of endogenous AHR ligands. Indeed, certain commensal bacteria, for example *Lactobacillus reuteri*, can produce tryptophan-derived AHR ligands that suppress inflammatory immune responses in the gut. Likewise, also pathogens, including *Mycobacterium tuberculosis,* seem to produce immunosuppressive AHR ligands as an immune escape mechanism [11]. Along the gut-liver axis, such AHR ligands produced by gut microbiota might reach the liver via the portal blood and shape immune responses locally in the liver. In light of the hypothesis that intestinal dysbiosis might be a key driver in the pathogenesis of fatty liver disease, a recent study by Krishnan et al. analyzed the metabolite profile of germ-free versus conventionally housed mice fed a low- or high-fat diet (HFD). Interestingly, they found decreased levels of the tryptophan-derived AHR ligands tryptamine and indole-3-acetate (I3A) in HFD-fed mice. Mechanistically, these AHR ligands attenuated the inflammatory TNF-α, IL-1β, and monocyte chemoattractant protein-1 response of macrophages following stimulation with LPS and palmitate acid, mimicking two typical insults in NAFLD. Moreover, I3A restricted the expression of the AHR-regulated lipogenesis enzymes fatty acid synthase (Fasn), and the cholesterol metabolism regulator sterol regulatory element-binding protein-1c (SREBP-1c) in hepatocytes. Therefore, depletion of gut-derived AHR ligands seems to be a major determinant for the progression of NAFLD [48]. Accordingly, in alcohol-induced liver injury, abundance of microbiota-derived tryptophan metabolites that signal through AHR as well as treatment with the AHR ligands FICZ [49] or I3C [50] reduced liver pathology. Conversely, *Ahr* knockout mice showed increased susceptibility to ethanol-induced liver damage. In patients, alcoholic liver disease was associated with low levels of intestinal microbiota-derived tryptophan metabolites, underscoring the clinical impact of the AHR pathway in alcoholic liver disease [49]. Interestingly, the alteration in gut microbiota composition has also been implicated as a risk factor for exacerbated acetaminophen-induced acute liver injury [51]. Although here, the underlying molecular mechanisms were not focus of the study, it is likely that dysbiosis was accompanied with changes in gut bacteria-derived metabolites, including AHR ligands. Accordingly, in our recent study, we demonstrated that the tryptophan derivative ITE that can be produced in the gut strongly exacerbated acetaminophen (APAP)-induced hepatotoxicity by directly activating AHR and the APAP-metabolizing enzyme CYP1A2 in hepatocytes [52]. Therefore, endogenous AHR ligands that can be produced by the intestinal microbiome might increase susceptibility to APAP hepatotoxicity. Note that presumably depending on the underlying pathogenic mechanisms, hepatic AHR activation can have both beneficial effects, as described in alcohol-induced liver injury [49, 50], and detrimental effects, as shown for acetaminophen-induced acute liver injury [52].

Moreover, besides the well-described AHR ligands derived from tryptophan, microbiota-derived short chain fatty acids (SCFA) that induce lL-22 production in the gut also seem to activate AHR [53]. Although the role of SCFA in hepatic immune responses has not been elucidated yet, it is tempting to speculate that SCFA might also activate AHR in the liver and thereby influence hepatic immune homeostasis.

## AHR in liver fibrosis and NASH

Already in 1995, Fernandez-Salguero et al. described hepatic fibrosis in *Ahr* knockout mice, which appeared to be rather mild and confined to the bile ducts [28]. However, the role of AHR in liver fibrosis remains controversial, as both loss and exacerbation of AHR activity seem to induce liver fibrosis [29, 54–57]. In an elegant study, using wild-type or conditional *Ahr* knockout mice that lacked *Ahr* in hepatocytes, Kupffer cells, or stellate cells, Yan et al. dissected the respective contribution of AHR activation in different liver cell types to fibrosis development in mouse models of CCl4-induced fibrosis or bile duct ligation. They found that *Ahr*-deficient HSCs were more sensitive to activation, whereas treatment with AHR ligands suppressed TGFβ-induced murine and human HSC activation. Most interestingly, specific disruption of *Ahr* in HSC but not other liver cells was causative for enhanced fibrogenesis. Moreover, in the CCl4 model, application of ITE could prevent fibrosis induction, suggesting AHR in HSCs as a potential target for the treatment of liver fibrosis [54]. In support of this notion, application of the AHR ligand 3′3-diindolylmethane (DIM), a metabolite of I3C, was likewise protective in murine liver fibrosis. Yet, in this study, AHR signaling was not directly addressed [55]. However, in another study, an adverse effect of AHR in liver fibrosis has been observed. Herein, the authors show a shifted balance towards more IL-17+ CD4+ T cells and less FOXP3high Tregs as well as increased IL-17 and IL-22 production in advanced fibrosis. Blocking of IL-22 production by administration of an AHR antagonist inhibited fibrogenesis in mouse models of liver fibrosis. In conclusion, AHR seems to be an important mediator of type 3 inflammation in the course of liver fibrogenesis that is mainly mediated by IL-17 and IL-22 [56]. The role of AHR in CCl4-induced acute liver injury and liver fibrosis had been further addressed by Hoshi et al., showing that blockade of AHR signaling via inhibition of the kynurenine-producing enzyme IDO2 attenuated acute liver damage and also liver fibrosis upon repeated CCl4 application [57]. The observed discrepancies regarding the role of AHR in liver fibrogenesis might at least in part be explained by different experimental approaches. Indeed, Hoshi et al. focused specifically on the AHR ligand kynurenine and IDO2, while Yan et al. used conditional *Ahr* knockout mice and the high-affinity AHR ligands ITE and TCDD in their study. Taken together, the role of AHR in liver fibrogenesis seems rather complex and is only beginning to be understood.

In the last couple of years, several studies addressing AHR function in nonalcoholic steatohepatitis (NASH) and nonalcoholic fatty liver disease (NAFLD) have emerged. Making use of mice with constitutive or defective AHR signaling, Lee et al. investigated the contribution of AHR to steatosis development. Interestingly, they identified CD36 as a new AHR target and linked steatosis-promoting AHR activation to increased expression of CD36 and lipid uptake into liver cells via CD36. Therefore, the authors suggest AHR and CD36 as novel therapeutic targets in fatty liver disease [58]. Based on the putative NAFLD-promoting role of AHR, Xia et al. tested the AHR inhibitor alpha-naphthoflavone (ANF) as therapeutic regimen in HFD-fed mice. They found that ANF treatment indeed reduced the expression of *Ahr* and the downstream molecules Cyp1a1 and Tnf-α, leading to reduced oxidative stress, insulin resistance, and attenuation of NAFLD [59]. Accordingly, the pro-steatotic effect of AHR via CYP1A1 activation had been further linked to estrogen metabolism, as estrogen degradation by CYP1A1 diminished the known protective effect of 17β-estradiol (E2) on steatosis [60]. In a study using mice with constitutive expression of human *AHR* specifically in the liver, fibroblast growth factor 21 (FGF21) could be identified as a direct AHR target and a major mediator of both hepatic steatosis and systemic insulin hypersensitivity [61]. However, protective effects of AHR in hepatic steatosis have also been suggested. As described above, gut microbiota–derived AHR ligands can attenuate inflammatory macrophage responses and lipogenesis in hepatocytes [48]. Likewise, high fat diet-fed mice with liver-specific *Ahr* knockout displayed severe steatosis and liver inflammation owed to up-regulated de novo lipogenesis. Lipotoxicity was linked to decreased hepatic expression of the newly identified AHR target gene suppressor of cytokine signal 3 (*Socs3*) [62]. Moreover, in order to reverse the shifted Th17/Treg balance in NASH, the AHR ligand DIM was tested in methionine-choline-deficient (MCD)-diet fed mice. Notably, DIM administration reversed the Th17/Treg imbalance to a Treg predominance, resulting in attenuation of hepatic steatosis and inflammation [63].

## AHR in hepatocellular carcinoma

AHR is involved in carcinogenesis in various cancers, influencing all major stages of tumor development, i.e., initiation, progression, and metastasis formation. Whereas the exogenous AHR ligand dioxin has long been recognized as carcinogen, recent progress has been made in determining the relevance of endogenous AHR ligands that seem to significantly contribute to tumor immune escape. Of note, AHR ligands contributing to an immunosuppressive niche can be produced by the host tissue but also by the tumor itself. Indeed, tumor cells and infiltrating immune cells such as DCs and macrophages can up-regulate the kynurenine producing enzymes TDO and IDO1. In conjunction with increased AHR expression, this can result in sustained AHR activation and perpetuation of a pro-tumorigenic immunosuppressive microenvironment [64].

In diethylnitrosamine (DEN)-induced hepatocarcinogenesis, *Ido* knockout mice showed significantly less tumor burden than their wild-type counterparts, which overexpressed IDO and L-kynurenine in hepatocellular carcinoma (HCC) as compared to surrounding tissue. The protection of *Ido* knockout mice was associated with an increased expression of CD8, as well as cytotoxicity-related genes, including granzyme B and perforin in hepatic tumors [65]. In HCC, Wang et al. recently deciphered an important molecular circuit in which the proto-oncogene intestine specific homeobox (ISX) induced IL-6-dependent up-regulation of TDO and IDO1. The resulting increased kynurenine/AHR signaling further promoted ISX expression. Notably, this positive feedback loop resulted in increased tumor cell proliferation, up-regulation of the immune checkpoint molecule PD-L1, and an impaired CD8+ T cell response, partly explaining tumor immune escape in HCC [66]. In line with these findings, expression of AHR, together with IDO1, kynurenine, and PD-L1, correlated with poor prognosis in hepatocellular carcinoma patients [66]. Tissue microarray analysis of 153 HCC patients confirmed a negative correlation of IDO expression and overall survival [67]. Likewise, as a promoter of HCC proliferation and tumor invasion, TDO has also been suggested as a new prognostic biomarker of HCC [68].

Whereas the role of AHR in establishing an immunosuppressive tumor niche is well recognized, it remains unclear, whether AHR activation in hepatic progenitor cells might impact on liver carcinogenesis. Indeed, there are some reports by the group of Vondráček et al., showing that AHR ligands affect several potentially tumor-promoting processes and signaling pathways in liver progenitor cells, such as Wnt/β-catenin signaling [69–71]. Likewise, Moreno-Marín et al. suggested that AHR-mediated regulation of stem-like liver cell expansion and pluripotency is an important control mechanism of liver regeneration and suppression of carcinogenesis [72]. Note, however, that these studies were mainly conducted in cell culture models and that the general in vivo relevance of hepatic progenitor cells in liver injury and regeneration is still highly controversial [73].

## AHR as therapeutic target in liver disease

The manifold functions of AHR in regulating inflammatory, fibrogenic, or tumorigenic processes in the liver suggest AHR as a promising therapeutic target in various liver diseases. Given that in several mouse models of autoimmune disease, including experimental autoimmune encephalomyelitis, diabetes, or psoriasis, endogenous non-toxic AHR ligands have already been successfully tested as potent immunosuppressive agents [12, 16, 17], it is conceivable that such AHR ligands might also be of therapeutic value in autoimmune diseases targeting the liver. In support of this notion, AHR dysfunction has been recently linked to autoimmune hepatitis [41]. Likewise, although so far only demonstrated in mice, attenuation of the acute phase response by dietary AHR ligands [47], prevention of liver fibrosis by application of ITE [54], or of alcohol-induced liver injury by FICZ [49] further suggest AHR ligands as new therapeutic regimens in liver disease. Vice versa, in liver infection, inhibition of AHR might boost anti-infectious immunity, as shown in *Trypanosoma cruzi* infection [46]. Moreover, targeting the IDO1/TDO2-kynurenine-AHR pathway to restore potent anti-tumor immune responses is an important goal of cancer immunotherapy [74, 75], which might be highly relevant for HCC and cholangiocarcinoma therapy.

Of note, knowledge about the kinetics of AHR ligand activity is an important prerequisite for therapeutic application. On the one hand, sufficient bioavailability is important for effectiveness; on the other hand, persistence of AHR ligand binding might have adverse effects [76]. Moreover, there is accumulating evidence that many AHR ligands exert their functions in an organ- or cell-specific manner [76–78] Table 2, offering the opportunity for the selection of specific AHR ligands or the design of tailor-made synthetic AHR agonists or antagonists according to the respective therapeutic indication [77, 78]. However, a major caveat for the pharmaceutical use of such selective AHR modulators remains the limited predictability of their in vivo function, requiring extensive testing in order to prevent unwanted side effects [77, 78].

## Concluding remarks

**Fig. 2:**
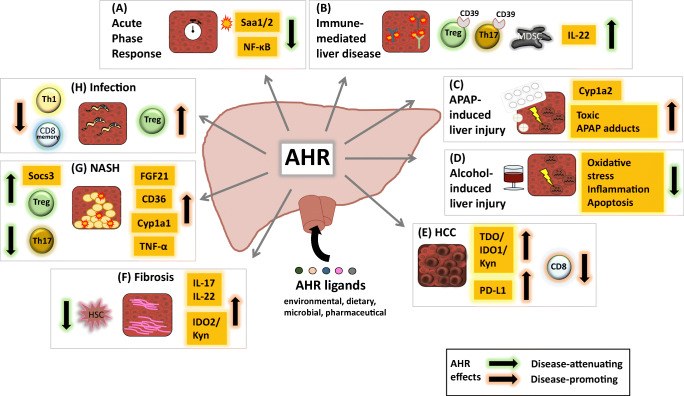
Functional role of AHR in liver disease. AHR activation can promote or dampen liver disease pathogenesis, as indicated by red or green arrows, respectively. AHR activating ligands can derive from various endogenous or exogenous sources or can be produced in the liver itself (see Table 1). (**A**) Acute phase response: AHR activation impairs NF-κB-mediated expression of acute phase genes such as Saa1/2 [47]. (**B**) Immune-mediated liver disease: anti-inflammatory CD39 expression on Treg or Th17 cells is AHR dependent [41]. AHR-mediated induction of suppressive MDSCs [38]. AHR controls protective IL-22 expression in ILCs and CD4+ T cells [34, 35, 37]. (**C**) APAP-induced liver injury: AHR activation induces the APAP-metabolizing enzyme CYP1A2, resulting in increased hepatotoxicity [52]. (**D**) Alcohol-induced liver injury: AHR activation reduces EtOH-induced oxidative stress, inflammation, and hepatocyte apoptosis [49, 50]. (**E**) HCC: increased production of the AHR ligand kynurenine via TDO and IDO1 results in upregulation of PD-L1, impaired CD8 T cell responses, and tumor progression [65–68]. (**F**) Fibrosis: AHR-dependent IL-17 and IL-22 production as well as AHR activation via IDO2/Kyn can promote liver fibrosis [56, 57], while ITE-induced AHR activation in HSCs dampens liver fibrosis [54]. (**G**) NASH: AHR-induced CD36 [58], FGF21[61], as well as the AHR downstream molecules Cyp1a1 and TNF-α [59] promote NASH. Vice versa, AHR-induced Socs3 [62] and AHR-dependent induction of a Treg versus Th17 predominance [61] attenuate NASH. (**H**) AHR restricts anti-infectious immunity in *Trypanosoma cruzi* infection by promoting Tregs and inhibiting Th1 responses and CD8 T cell memory development [46]

Although we are only beginning to understand the complex role of AHR in hepatic homeostasis and disease, it has become unequivocally clear that AHR is an important regulator of metabolic and immunological processes in the liver (Fig. 2). Whereas in immune-mediated liver disease, the current literature rather suggests an immunosuppressive function of AHR, the role of AHR in liver fibrosis or NASH remains ambiguous. Indeed, there is evidence for both beneficial and detrimental effects of AHR signaling, most likely depending on the respective experimental setup and the presence of contextual signals. Likewise, while the outcome of AHR activation in acute acetaminophen-liver injury is exacerbation of liver damage, AHR ligands seem to have beneficial effects in ethanol-induced liver injury. Therefore, in order to develop AHR-based therapeutic strategies for liver disorders, a thorough understanding of AHR function in the respective disease-driving pathways is required. Moreover, given that AHR is widely expressed throughout the body and has pleiotropic functions, targeting strategies for specific delivery of AHR agonists or antagonists to the relevant target cells, such as HSCs in liver fibrosis [79] or APCs in immune-mediated diseases [80], are highly desirable. Of note, most of the studies assessing the therapeutic potential of AHR ligands have been conducted in rodents. Yet, human and rodent AHR differ considerably in ligand selectivity [64], indicating that murine studies might under- or overestimate the therapeutic efficacy of specific ligands for potential application in patients. Therefore, future studies in humanized mice and patients are needed to further explore AHR-based therapies in liver disease.
